# Comparison of different T2* sequences with and without parallel imaging for the assessment of iron overload

**DOI:** 10.1186/1532-429X-16-S1-P259

**Published:** 2014-01-16

**Authors:** Juliano L Fernandes, Paulo H de Souza, Fernanda B Pinto, Andreas Greiser, Ralph Strecker, Luciana A Fioravante, Jose Alvaro da Silva, Gabriel Figueiredo, Jose M Kalaf, Otavio R Coelho

**Affiliations:** 1 University of Campinas, Campinas, SP, Brazil; 2 Instituto de Ensino e Pesquisa Jose Michel Kalaf, Campinas, Brazil; 3 Siemens Healthcare, Erlangen, Germany; 4 Siemens Ltda, Sao Paulo, Brazil

## Background

Iron overload assessment using cardiovascular magnetic resonance (CMR) imaging has become the gold standard diagnostic method for this condition. Because patients usually need to undergo multiple exams throughout their lifetime it is important to assess if the myocardial and liver iron concentrations measured can be accomplished interchangeably using different techniques. We sought to examine whether different types of T2* sequences in the heart generate similar results and whether the use of parallel imaging in addition to each sequence (both in the heart and liver) would interfere in the final values obtained.

## Methods

Eighty five patients undergoing regular transfusions underwent CMR imaging for the assessment of iron overload at 1.5T using a ROI-based T2* method. In the same exam, a multi-echo GRE single short-axis mid ventricular slice of the heart was obtained using the following sequences: (1) black-blood acquisition in diastole (BBD); (2) bright-blood acquisition in systole (BrBS); (3) bright-blood acquisition in diastole (BrBD). All sequences were obtained once with parallel imaging (R = 2) and again without it completing 6 different sets. A single multi-echo axial slice of the liver was also imaged twice with and without parallel imaging. All results were compared using repeated measures ANOVA (with Bonferroni correction for pairwise comparisons) and Bland Altman analysis.

## Results

The mean age of the patients was 28.7 ± 15.2 years with 49/85 (58%) men. No differences were observed among all the six variations of T2* sequences taken together (P = 0.13) and in pairwise comparisons: BBD non-parallel T2* of 28.1 ± 10.2 ms; BBD parallel 28.9 ± 12.0 ms; BrBS non-parallel 29.3 ± 11.7 ms; BrBS parallel 29.9 ± 12.1 ms; BrBD non-parallel 29.4 ± 11.9 ms; BrBD parallel 29.4 ± 12.3 ms. The T2* values for the liver using non-parallel imaging and parallel imaging were also similar with 6.6 ± 6.1 ms and 6.5 ± 6.2 ms, P = 0.97. Using the BBD non-parallel T2* as the reference for the heart, Bland-Altman analysis showed a mean difference of -0.93 ms (-1.8 to -0.02 95%CI) to the BBD parallel sequence, -1.3 ms (-2.3 to -0.16) to the BrBS non-parallel sequence, -1.8 ms (-3.2 to -0.4) to the BrBS parallel sequence, -1.3 ms (-2.5 to -0.17) to the BrBD non-parallel sequence and -1.3 ms (-2.5 to -0.16) to the BrBD parallel sequence. For the liver, the mean difference among parallel and non-parallel images was -0.09 ms (-0.17 to -0.008 95% CI). Parallel imaging in the heart and in the liver allowed for a mean reduction of acquisition time of 42% (range 32 - 47%).

## Conclusions

Different T2* sequences can be used to achieve similar measurements of iron overload both in the heart and liver. Moreover, the use of parallel imaging does not significantly interfere with the results allowing for faster acquisition without compromising the final values.

## Funding

Fundacao de Amparo a Pesquisa do Estado de Sao Paulo (FAPESP).

